# Defining Sarcopenia in Oncology by CT-Based Muscle Mass: The Clinical and Research Consequences of a Diagnostic Surrogate

**DOI:** 10.3390/diagnostics16111611

**Published:** 2026-05-25

**Authors:** Erkan Topkan, Efsun Somay, Duriye Ozturk, Ugur Selek

**Affiliations:** 1 Department of Radiation Oncology, Faculty of Medicine, Baskent University, 01120 Adana, Turkey; docdretopkan@gmail.com; 2 Department of Oral and Maxillofacial Surgery, Faculty of Dentistry, Baskent University, 06490 Ankara, Turkey; 3 Department of Radiation Oncology, School of Medicine, Afyonkarahisar Health Sciences University, 03030 Afyonkarahisar, Turkey; duriyeozturk07@gmail.com; 4 Department of Radiation Oncology, School of Medicine, Koc University, 34010 Istanbul, Turkey; ugurselek@yahoo.com

**Keywords:** sarcopenia, myopenia, cancer cachexia, muscle strength, computed tomography, treatment toxicity, risk stratification, supportive oncology

## Abstract

Sarcopenia is increasingly invoked as a determinant of treatment-related toxicity, perioperative morbidity, treatment intolerance, and survival in oncology; however, contemporary international consensus frameworks define sarcopenia as a multidimensional neuromuscular syndrome centered on impaired muscle strength, physical performance, and muscle quality, whereas most oncologic studies operationalize sarcopenia using computed tomography (CT)-derived skeletal muscle mass alone. In this context, muscle quantity is effectively employed as a diagnostic surrogate for a function-centered syndrome. CT-defined skeletal muscle depletion—more precisely described as myopenia—remains a reproducible and clinically informative structural biomarker, yet defining sarcopenia by muscle mass alone aggregates biologically heterogeneous phenotypes, including neuromuscular dysfunction, inflammation-driven cachexia, and substrate-related malnutrition. Such surrogate-based definitions contribute to variable prevalence estimates, inconsistent prognostic associations, and interpretive instability across studies. Clinically, reliance on CT-based muscle mass as a surrogate for sarcopenia may influence chemotherapy dosing, perioperative risk stratification, and supportive care allocation without direct assessment of neuromuscular function; in research settings, mass-based definitions may dilute treatment effects in exercise or nutritional trials and complicate meta-analytic synthesis by conflating structural and functional constructs. This analysis does not question the value of radiologic muscle assessment but argues that CT-derived muscle mass should be recognized as a structural biomarker within a multidimensional framework rather than as a standalone diagnostic surrogate for sarcopenia. A tiered, oncology-adapted approach integrating functional assessment, muscle quality, and relevant metabolic context may enhance risk discrimination, improve trial design, and strengthen translational precision in supportive oncology.

## 1. Introduction

Sarcopenia is a progressive disorder of skeletal muscle, manifesting as reductions in muscle strength, physical performance, and/or muscle mass, and conferring heightened risks of functional impairment, disability, treatment intolerance, and mortality. Originally conceptualized within the domain of geriatric medicine, sarcopenia has since been recognized as a clinically salient construct across a spectrum of chronic disease states, notably within the field of oncology [1]. Among patients with cancer—particularly those affected by gastrointestinal, thoracic, or head-and-neck malignancies—sarcopenia is highly prevalent and is frequently exacerbated by tumor progression, systemic inflammation, malnutrition, metabolic dysregulation, and the adverse effects of treatment [1,2,3,4,5,6,7,8]. In the oncologic setting, sarcopenia is strongly associated with increased susceptibility to chemotherapy- and radiotherapy-induced toxicity, postoperative complications, unplanned hospitalizations, treatment interruptions, and inferior survival outcomes, thereby underscoring its role as a critical marker of physiologic reserve and oncologic vulnerability [4,5,6,7,8].

Over the past decade, the conceptualization of sarcopenia has undergone pronounced evolution. Initial frameworks—such as those proposed by the European Working Group on Sarcopenia in Older People 1 (EWGSOP1), the International Working Group on Sarcopenia (IWGS), and the Foundation for the National Institutes of Health Sarcopenia Project (FNIH)—incorporated various combinations of muscle mass, strength, and physical performance into their diagnostic algorithms [1,9,10]. More recent consensus statements, including EWGSOP2, AWGS2, SDOC, and the Global Leadership Initiative on Sarcopenia (GLIS), have shifted emphasis toward muscle strength, physical performance, muscle quality, and comprehensive multidomain assessment [2,3,11,12]. Collectively, these frameworks represent a decisive transition from morphology-centric to function-oriented definitions of sarcopenia, reflecting a more nuanced and patient-centered approach.

Notwithstanding these conceptual advances, oncologic research has largely continued to operationalize sarcopenia via radiologic morphometric quantification of skeletal muscle mass. Computed tomography (CT)-derived assessments at the third lumbar vertebral level—encompassing indices such as the skeletal muscle index (SMI), psoas muscle index (PMI), and total psoas area (TPA)—remain the most extensively employed and technically validated imaging proxies for whole-body skeletal muscle mass in oncology populations [13,14,15,16]. It is important to note, however, that such validation predominantly pertains to anatomic reproducibility and prognostic correlation, rather than affirmation of sarcopenia as a multidimensional neuromuscular syndrome. As a result, CT-defined skeletal muscle depletion may not adequately capture deficits in muscle strength, physical performance, or muscle quality, all of which are now recognized as central to contemporary consensus definitions.

Alternative imaging modalities—including cervical and thoracic vertebral muscle assessment and magnetic resonance imaging–based evaluation of muscle quality and myosteatosis—have broadened the morphologic characterization of body composition in oncology. Nonetheless, these techniques remain principally focused on structural rather than functional domains of muscle health. This distinction bears significant clinical relevance, as decrements in muscle strength and neuromuscular performance may precede overt losses in muscle mass and are frequently more predictive of adverse outcomes than morphologic parameters alone [9,10,17]. Persistent reliance on morphologic surrogates, despite the emergence of function-centric definitions, has consequently engendered considerable heterogeneity in reported sarcopenia prevalence, inconsistent prognostic estimates, and interpretive variability across oncologic investigations [18,19,20,21].

Importantly, the present analysis does not seek to diminish the established prognostic value or clinical utility of radiologic skeletal muscle assessment. CT-derived muscle depletion continues to serve as a reproducible, scalable, and clinically meaningful structural biomarker within oncology. Nevertheless, defining sarcopenia solely on the basis of muscle quantity effectively conflates morphology with a multidimensional neuromuscular syndrome, thereby aggregating biologically disparate phenotypes, such as functional sarcopenia, inflammation-driven cachexia, and malnutrition-related muscle depletion. This conceptual conflation has important implications for risk stratification, allocation of supportive care resources, interpretation of therapeutic outcomes, and the design of interventional trials.

Accordingly, a critical reassessment of the conceptual and diagnostic operationalization of sarcopenia within the oncologic context is warranted. The present review synthesizes the evolution of contemporary sarcopenia frameworks, scrutinizes the methodological ramifications of predominantly CT-based muscle mass definitions in oncology research, and explores pragmatic, multidimensional strategies that may enhance translational precision, phenotypic interpretation, and the implementation of supportive oncology assessment strategies [1,2,3,4,5,6,7,8,9,10,11,12,18,19,20,21].

## 2. Literature Identification and Scope

This article was conceived as a narrative and conceptual review rather than a systematic or scoping synthesis. The relevant literature was identified through targeted searches of PubMed/MEDLINE, Scopus, and Google Scholar, with a focus on publications addressing sarcopenia consensus definitions, CT-derived skeletal muscle assessment, myopenia, muscle strength and quality, cancer cachexia, malnutrition, and oncologic body composition research. Priority was given to international consensus statements, foundational methodological frameworks, meta-analyses, and representative oncology studies evaluating radiologic skeletal muscle metrics in relation to treatment tolerance, treatment-related toxicity, postoperative outcomes, and survival endpoints. The resulting synthesis was intentionally interpretive and illustrative rather than exhaustive or systematically quantitative, with the principal aim of elucidating the conceptual and clinical implications of operationalizing sarcopenia in oncology through imaging-derived muscle mass metrics alone.

## 3. Conceptual and Diagnostic Frameworks of Sarcopenia

**Figure 1:**
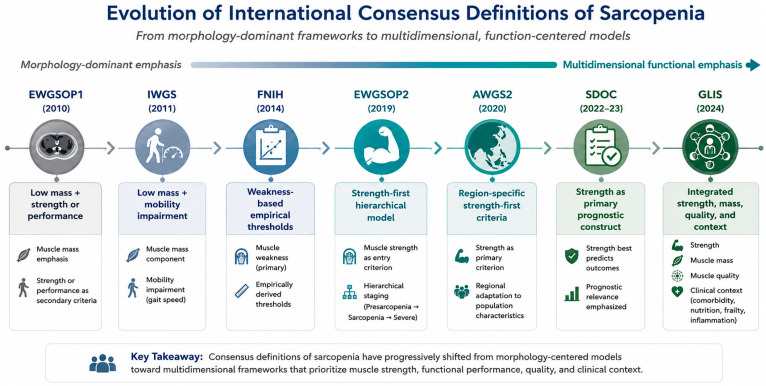
Evolution of consensus definitions of sarcopenia from 2010 to 2024, illustrating the shift from morphology-oriented criteria emphasizing low muscle mass toward strength-centered and multidimensional frameworks. Early definitions combined muscle mass with strength or performance measures, whereas later statements prioritized muscle strength and incorporated hierarchical and integrated diagnostic models.

To contextualize the clinical relevance of sarcopenia in oncology, it is essential to examine the conceptual and diagnostic frameworks that have shaped its definition over time. Over the past two decades, sarcopenia has evolved from a morphology-centered construct focused primarily on muscle mass into a multidimensional clinical syndrome emphasizing neuromuscular function, physical performance, and, more recently, muscle quality (Figure 1). This evolution has been driven by accumulating evidence that functional impairments, rather than isolated anatomic deficits, are more closely linked to adverse clinical outcomes [2,11,20,22,23,24,25,26,27,28,29,30]. Table 1 summarizes the main international consensus definitions and diagnostic frameworks that support this change. These include those put forward by the EWGSOP1, IWGS, FNIH, EWGSOP2, AWGS, SDOC, and GLIS. The table compares their diagnostic priorities, core components, and key conceptual advances. The following sections review these frameworks in chronological order to highlight how shifts in diagnostic emphasis have shaped contemporary assessment paradigms and informed ongoing debates in oncology sarcopenia research.

### 3.1. Foundational Tripartite Model (EWGSOP1, 2010)

The 2010 publication of the EWGSOP1 report marked a seminal milestone by providing the first widely adopted clinical definition of sarcopenia and establishing a conceptual foundation for subsequent research [1]. Sarcopenia was defined as the coexistence of low skeletal muscle mass with either reduced muscle strength or impaired physical performance, thereby introducing a tripartite framework integrating both structural and functional domains. This model was supported by emerging evidence demonstrating that reductions in appendicular lean mass and gait speed independently predicted disability, institutionalization, and mortality among older adults [31,32].

Importantly, EWGSOP1 acknowledged that muscle atrophy and functional decline do not invariably occur in parallel. Some individuals may exhibit reduced muscle mass despite preserved strength and performance, whereas others demonstrate clinically meaningful weakness despite relatively preserved muscle quantity. Accordingly, the framework was designed to identify individuals at elevated clinical risk rather than those with isolated morphologic variation.

One of the major contributions of EWGSOP1 was the introduction of a pragmatic stepwise diagnostic algorithm in which gait speed served as an initial screening tool followed by confirmatory muscle mass assessment using DXA or CT imaging [1]. This methodological approach substantially influenced subsequent research methodology and clinical screening strategies. Nevertheless, important limitations soon became apparent. Overdiagnosis occurred among individuals with constitutionally low lean mass or smaller body habitus, whereas under-recognition was observed in patients with obesity-related myosteatosis and impaired muscle quality despite relatively preserved muscle mass [2,20].

In oncology populations, an additional mismatch emerged: CT-derived skeletal muscle metrics were routinely available, whereas standardized functional assessments were rarely collected, particularly in retrospective cohorts. Consequently, muscle quantity increasingly became operationalized as a surrogate diagnostic proxy for sarcopenia, contributing to substantial heterogeneity in reported prevalence estimates and prognostic associations across oncologic studies [4]. These limitations ultimately prompted the development of subsequent frameworks that placed progressively greater emphasis on functional assessment, muscle quality, and population-specific interpretation.

### 3.2. Transition Toward Mobility and Empiric Thresholds (IWGS, 2011 and FNIH Sarcopenia Project, 2014)

The IWGS, convened in 2011, sought to address important limitations of the EWGSOP1 framework, particularly its relative underemphasis of mobility as a major determinant of functional independence and health-related outcomes [9,31]. By prioritizing gait speed as the principal criterion for case identification while maintaining low appendicular lean mass as a diagnostic component, the IWGS reconceptualized sarcopenia as fundamentally a mobility-centered disorder. This paradigm shift redirected conceptual emphasis from isolated structural deficits toward functional decline.

This mobility-centered perspective is particularly relevant in oncology. Among individuals with head-and-neck or thoracic malignancies, neuromuscular dysfunction related to radiotherapy, chemotherapy, systemic inflammation, or neuropathic injury may impair gait speed before measurable skeletal muscle loss becomes apparent [33]. These observations highlight a major strength of the IWGS framework: its ability to identify clinically meaningful functional decline preceding overt morphologic change. Nevertheless, reliance on mobility alone also introduced limitations, as patients with sarcopenic obesity or metabolically complex disease states may retain preserved gait speed despite substantial muscle weakness and impaired muscle quality [20,34].

The FNIH Sarcopenia Project, introduced in 2014, further advanced the field by establishing empirically derived diagnostic thresholds linking muscle strength and lean mass to clinically relevant mobility impairment [10]. Importantly, the FNIH framework demonstrated that muscle weakness frequently represents the earliest and most clinically meaningful manifestation of sarcopenia, whereas muscle mass alone explains only a limited proportion of functional variability. These findings further reinforced the growing recognition that functional impairment—rather than isolated morphologic reduction—constitutes the central pathophysiologic domain of sarcopenia.

Although questions regarding generalizability to populations with differing body composition, ethnic background, and cancer-related metabolic alterations persisted [10,35,36], the IWGS and FNIH frameworks collectively accelerated the transition toward function-centered conceptualization of sarcopenia and strengthened the rationale for prioritizing muscle strength and performance within subsequent diagnostic paradigms.

### 3.3. Strength-Centered and Quality-Informed Redefinition of Sarcopenia (EWGSOP2, AWGS2, SDOC, and GLIS)

Building on previous conceptual frameworks, the European Working Group on Sarcopenia in Older People 2 (EWGSOP2), published in 2019, redefined sarcopenia as a muscle disease primarily characterized by reduced skeletal muscle strength [2]. The revised framework introduced a staged diagnostic algorithm in which low muscle strength serves as the entry criterion, muscle mass provides confirmatory evidence, and impaired physical performance reflects disease severity. This framework further reinforced function-centered assessment aligned with clinically meaningful outcomes. Similarly, the Asian Working Group for Sarcopenia 2 (AWGS2) adopted a strength-first approach while refining thresholds to better reflect regional differences in body composition, functional capacity, and epidemiologic characteristics, thereby reducing misclassification across diverse populations [3].

The Sarcopenia Definitions and Outcomes Consortium (SDOC) further reinforced this conceptual shift through pooled analyses demonstrating that grip strength consistently outperformed muscle mass indices in predicting mobility limitation, disability, hospitalization, and mortality, whereas muscle mass contributed only limited incremental prognostic value after accounting for strength [11]. These findings strongly supported a “strength-first, mass-optional” paradigm, an approach particularly relevant in oncology, where CT-derived estimates of muscle area demonstrate inconsistent associations with treatment tolerance and clinical outcomes. Moreover, systemic inflammation, metabolic dysregulation, and neuromuscular dysfunction frequently impair muscle quality and strength before measurable skeletal muscle loss becomes apparent in patients with cancer [4,5,15,17,20,21].

The Global Leadership Initiative on Sarcopenia (GLIS), convened in 2024, integrated these evolving perspectives into a harmonized international framework while preserving flexibility for regional and clinical variation [12]. Importantly, the GLIS distinguished diagnostic constructs—including muscle strength, muscle mass, and muscle quality—from prognostic constructs such as disability, frailty progression, treatment toxicity, and mortality. This distinction helps avoid circular reasoning in which sarcopenia is defined solely through low muscle mass and subsequently validated by demonstrating that muscle mass predicts adverse outcomes.

A major contribution of the GLIS was the formal incorporation of muscle quality as a central diagnostic domain, particularly through CT- and MRI-based assessment of myosteatosis. This refinement reflects growing evidence, especially in oncology, that fatty infiltration and reduced muscle density may carry greater prognostic significance than muscle mass alone and are strongly associated with frailty, treatment toxicity, postoperative complications, immunologic vulnerability, and mortality [20]. By integrating muscle quality alongside strength and muscle mass, the GLIS provides a framework that more accurately reflects the complex neuromuscular and metabolic disturbances characteristic of cancer and its treatments.

Collectively, the progressive evolution from the EWGSOP1 to GLIS illustrates a fundamental transformation in the conceptualization of sarcopenia. Contemporary frameworks increasingly conceptualize sarcopenia as a multidimensional neuromuscular syndrome rather than an isolated morphologic abnormality. This conceptual transition closely parallels broader shifts within oncology toward function-centered and physiologically informed assessment strategies that prioritize clinically meaningful vulnerability over isolated anatomic metrics.

### 3.4. Sarcopenia, Cachexia, and Malnutrition: Conceptual Overlap and Oncology-Specific Clinical Consequences

Sarcopenia, cachexia, and malnutrition are distinct yet frequently overlapping conditions that share the typical clinical manifestation of muscle loss but differ fundamentally in their underlying biology, clinical trajectories, and therapeutic implications [22,23] (Table 2). In oncology, failure to distinguish among these entities has become a significant source of diagnostic ambiguity, methodological heterogeneity, and potentially inappropriate clinical decision-making [24,25].

Sarcopenia is primarily a neuromuscular disorder characterized by progressive impairment in skeletal muscle strength and physical performance, with loss of muscle mass occurring as a secondary feature [2,9,11]. While age-related mechanisms predominate in primary sarcopenia, secondary sarcopenia may develop in association with chronic disease, inactivity, endocrine dysregulation, or treatment-related stress [1,2,3,36]. Importantly, sarcopenia is not inherently driven by systemic inflammation or negative energy balance and is often amenable, at least in part, to targeted interventions such as resistance exercise and structured nutritional support [2,11,17].

Conversely, cancer cachexia is a multifaceted metabolic syndrome induced by chronic systemic inflammation, tumor-derived catabolic signaling, and neuroendocrine dysregulation [22,23,24]. It is characterized by involuntary weight loss with preferential depletion of skeletal muscle mass, which may or may not be accompanied by loss of adipose tissue, and by limited responsiveness to conventional nutritional or pharmacologic interventions [22,23]. Muscle wasting in cachexia reflects accelerated proteolysis, mitochondrial dysfunction, and impaired anabolic signaling rather than disuse alone [24,26]. Functional decline is often rapid and profound, accompanied by fatigue, anorexia, and metabolic instability, and the condition is typically only partially reversible [22,23]. Clinically, cachexia frequently signifies advanced disease and markedly diminished physiologic reserve [4,5,6,23]. Importantly, cachexia represents a severe systemic state that may encompass all of the defining features of sarcopenia [22,25].

Malnutrition is a distinct but often coexisting condition, characterized by inadequate energy and protein intake relative to physiological requirements [26,27]. Muscle wasting in malnutrition is largely substrate-driven and may be reversible with timely and adequate nutritional rehabilitation [26]. Unlike cachexia, malnutrition is not necessarily accompanied by systemic inflammation, and unlike sarcopenia, it does not inherently reflect neuromuscular dysfunction [22,26]. However, prolonged malnutrition may precipitate or exacerbate sarcopenia, particularly in older adults and patients undergoing intensive cancer therapy [27,36].

In routine oncologic practice, these entities are frequently conflated due to reliance on radiologic morphometric surrogates, most commonly CT-derived muscle area [13,14,15,16,21]. Nevertheless, reduced skeletal muscle mass on imaging alone cannot reliably distinguish sarcopenia from cachexia or malnutrition, as all three conditions may present with low muscle mass [20,21,25]. This limitation is compounded by the routine availability of imaging data and the relative absence of functional testing, inflammatory markers, and systematic nutritional assessment in many oncologic datasets [18,25]. As a result, patients with cachexia or severe malnutrition are often labeled as “sarcopenic,” obscuring important biological distinctions and contributing to substantial variability in prevalence estimates and prognostic associations across studies [18,21,28].

The clinical implications of this misclassification become particularly evident when examined through an oncology-specific lens. In head-and-neck cancer, functional decline frequently precedes overt loss of muscle mass [33]. Radiotherapy, concurrent systemic therapy, cranial neuropathy, trismus, dysphagia, and pain can impair muscle strength and physical performance early in the disease course, even when whole-body muscle area appears relatively preserved [33]. Reliance on morphologic criteria alone may therefore underestimate sarcopenia prevalence in this population, while conflating treatment-related malnutrition or evolving cachexia—driven by mucositis, odynophagia, and reduced oral intake—with true neuromuscular sarcopenia [25,33]. Functional measures such as grip strength or performance-based assessments are often more sensitive indicators of vulnerability in this context [11,17].

In gastrointestinal malignancies, particularly pancreatic, gastric, and colorectal cancers, systemic inflammation, anorexia, and metabolic dysregulation are often prominent early features [23,24,29]. These patients commonly develop cancer cachexia, characterized by rapid, inflammation-driven muscle wasting that is poorly responsive to nutritional supplementation or exercise alone [22,23,24,29]. Labeling such patients as sarcopenic based solely on low muscle mass risks under-recognition of cachexia and may foster unrealistic expectations regarding reversibility [22,25]. Conversely, a subset of patients with gastrointestinal cancers—especially those with obesity or preserved body weight—may harbor severe muscle weakness, poor muscle quality, and marked myosteatosis despite apparently adequate lean mass, obscuring functional vulnerability when morphologic thresholds are used in isolation [20,25,34].

In thoracic malignancies, including lung and esophageal cancers, diagnostic ambiguity is further shaped by disease- and treatment-specific factors. Patients with lung cancer frequently exhibit chronic inflammation, hypoxemia, smoking-related comorbidities, and reduced physical activity, all of which contribute to early neuromuscular dysfunction independent of measurable muscle mass loss [24,28]. Declines in muscle strength and endurance may therefore precede radiologically manifest skeletal muscle loss, particularly in those receiving concurrent chemoradiotherapy or immunotherapy [7,28]. In esophageal cancer, profound nutritional compromise driven by dysphagia, odynophagia, and early satiety often leads to rapid weight loss and malnutrition before diagnosis [27,29]. Radiologic muscle loss in this setting does not reliably distinguish substrate deficiency from cachexia or sarcopenia, increasing the risk of inappropriate or incomplete intervention selection [25,26,27].

Across these oncologic contexts, misclassification carries tangible clinical consequences. Mislabeling cachexia or severe malnutrition as sarcopenia may result in overreliance on exercise-based interventions that are poorly tolerated or ineffective in the presence of active inflammation and metabolic instability [22,23,24]. Conversely, failure to recognize functional sarcopenia in patients with preserved muscle mass—such as those with sarcopenic obesity or pronounced myosteatosis—may delay appropriate supportive care and risk stratification [20,25,34]. From a research perspective, the conflation of these entities undermines the comparability of studies, inflates heterogeneity in prognostic estimates, and complicates the interpretation of interventional trials [18,21,28].

Contemporary consensus frameworks increasingly acknowledge these distinctions. Strength-centered definitions emphasize neuromuscular impairment as the defining feature of sarcopenia, while recent initiatives highlight the importance of separating diagnostic constructs (strength, mass, muscle quality) from prognostic outcomes [2,11,12]. In oncology, where inflammation, nutritional compromise, and treatment toxicity frequently coexist, such conceptual clarity is essential [25,28].

A more precise differentiation of sarcopenia, cachexia, and malnutrition—supported by integrated assessment of muscle strength, muscle quality, inflammatory burden, and nutritional status—is therefore critical for improving diagnostic accuracy, reducing misclassification, and guiding appropriate intervention selection [11,12,25]. Recognizing both the overlap and the distinctions among these conditions is central to advancing personalized cancer care, optimizing supportive strategies, and more accurately characterizing physiologic reserve and treatment vulnerability in patients with malignancy [4,5,6,28,29,30].

The following clinical vignettes illustrate how these conceptual distinctions manifest across standard oncologic settings and underscore the importance of integrated functional, morphologic, and metabolic assessment.

### 3.5. Clinical Vignette Box: Distinguishing Sarcopenia, Cachexia, and Malnutrition in Oncology Practice

#### 3.5.1. Vignette 1: Head-and-Neck Cancer (Functional Sarcopenia Predominant)

A 62-year-old man with locally advanced oropharyngeal squamous cell carcinoma undergoes definitive chemoradiotherapy. Baseline CT at the third cervical vertebral level demonstrates preserved skeletal muscle area. However, during treatment, he experiences progressive trismus, cranial neuropathy, and fatigue, along with a significant decrease in handgrip strength and gait speed. Despite stable body weight and minimal radiologic muscle loss, he experiences early treatment toxicity and prolonged recovery.

Interpretation: This presentation is consistent with functional sarcopenia driven by neuromuscular impairment rather than morphologic muscle loss. Reliance on CT-based muscle mass alone would underestimate vulnerability, whereas strength- and performance-based assessments better capture clinically relevant risk.

#### 3.5.2. Vignette 2: Gastrointestinal Cancer (Cancer Cachexia Predominant)

A 68-year-old woman with metastatic pancreatic adenocarcinoma presents with unintentional weight loss of 12% over three months, anorexia, elevated inflammatory markers, and a rapid decline in skeletal muscle area at the third lumbar vertebral level. Nutritional supplementation yields minimal improvement, and functional decline progresses despite preserved baseline physical performance.

Interpretation: The dominant process is cancer cachexia, characterized by inflammation-driven catabolism and resistance to nutrition alone. Labeling this patient as sarcopenic based solely on low muscle mass risks misclassification and may lead to inappropriate expectations regarding reversibility.

#### 3.5.3. Vignette 3: Thoracic Cancer (Malnutrition and Sarcopenic Obesity Overlap)

A 70-year-old man with distal esophageal carcinoma and obesity presents with dysphagia, early satiety, and reduced oral intake. CT imaging reveals a modest reduction in muscle mass with pronounced myosteatosis, whereas grip strength and chair-rise performance are markedly impaired.

Interpretation: This phenotype reflects overlapping malnutrition and functional sarcopenia masked by preserved body weight. Morphologic criteria alone would underestimate the risk, whereas an integrated assessment identifies substantial treatment vulnerability requiring aggressive nutritional rehabilitation and functional support.

## 4. The Persisting Radiology-Based Operationalization of Sarcopenia in Oncology

Despite the availability of multiple international consensus definitions conceptualizing sarcopenia as a multidimensional neuromuscular disorder, oncologic research and clinical practice have converged mainly on a single operational definition based on radiology-derived skeletal muscle loss. This operational convergence appears to reflect the practicality of methodological approaches and the routine availability of radiologic assessment, particularly CT, in oncology, rather than deliberate divergence from contemporary consensus frameworks [37]. As a result, what is labeled as “sarcopenia” in much of the oncologic literature represents a partial and incomplete surrogate of the true syndrome, capturing isolated morphologic muscle depletion while systematically omitting the functional and qualitative dimensions—muscle strength, physical performance, and muscle quality—that are central to modern consensus definitions [2,9,11,12]. This reductionist operationalization has been consistently observed across observational studies, pooled analyses, and meta-analyses in oncology and constitutes a significant source of diagnostic ambiguity and methodological heterogeneity within the field [4,5,6,18,21]. Importantly, this limitation is not confined to specific tumor entities but reflects a field-wide paradigm that applies broadly across solid malignancies. This divergence is most evident when compared with contemporary consensus definitions of sarcopenia.

### 4.1. From Multidimensional Syndrome to a Single Imaging Metric

**Figure 2:**
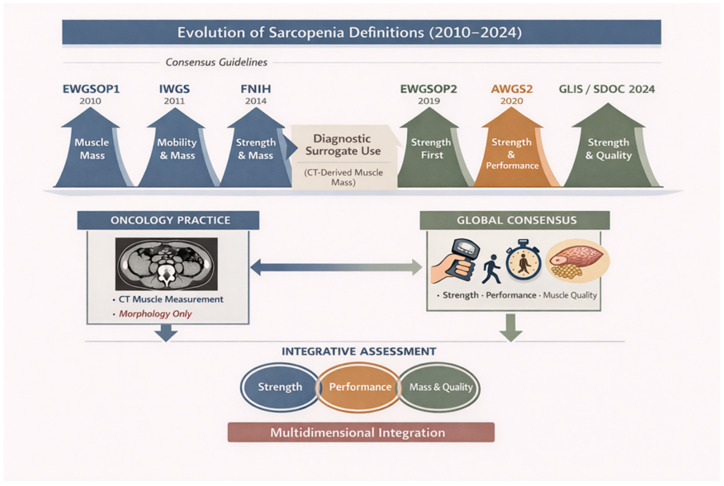
Operational differences between contemporary consensus definitions of sarcopenia and imaging-based practice in oncology. The schematic contrasts multidimensional consensus frameworks, in which muscle strength and performance constitute the primary diagnostic domain, with operational oncology practice that frequently applies CT-derived muscle mass as a diagnostic surrogate. The diagram illustrates how reliance on structural measures alone may aggregate biologically distinct phenotypes and influence prevalence estimates, risk stratification, and translational interpretation. Multidimensional integration, incorporating functional assessment and muscle quality, is presented as a harmonizing approach.

Contemporary consensus definitions of sarcopenia—including those proposed by the EWGSOP2, the SDOC, and the GLIS—conceptualize sarcopenia as a neuromuscular disorder in which muscle strength and function constitute the primary diagnostic determinants, with muscle mass serving a secondary or, in some frameworks, non-essential confirmatory role (Figure 2) [2,11,12]. Measures of physical performance and muscle quality further refine disease severity, functional impact, and clinical relevance. Notably, the SDOC framework does not require reduced skeletal muscle mass for a diagnosis of sarcopenia, underscoring the primacy of neuromuscular function over morphologic muscle loss [11].

Despite this clear functional emphasis, the dominant operationalization of sarcopenia in oncology remains almost exclusively morphologic, relying on radiology-derived skeletal muscle area or index—most commonly quantified on routine CT or MRI at the third lumbar vertebral level (L3) [13,14,15,16]. This imaging-centric approach implicitly collapses sarcopenia into a surrogate of muscle quantity, systematically bypassing the functional and qualitative dimensions that define sarcopenia as a clinical syndrome [38,39]. As a consequence, the term “sarcopenia” is frequently used in oncologic research to denote structural muscle loss, diverging from contemporary consensus definitions that define sarcopenia as a functionally defined neuromuscular disorder.

### 4.2. Why Radiologic Muscle Loss Became the Dominant Operational Definition

The widespread use of CT-derived skeletal muscle measurements has led to the operational definition of sarcopenia based primarily on muscle quantity in oncology studies [13,15]. Because CT imaging is routinely performed for staging, treatment planning, and surveillance across most solid malignancies, skeletal muscle measurements can be readily extracted from existing datasets without additional patient burden. This practical advantage has facilitated the rapid expansion of radiologic body composition research and has enabled large-scale retrospective analyses linking skeletal muscle depletion with adverse oncologic outcomes. However, this operational approach largely reflects pragmatic feasibility rather than strict adherence to contemporary consensus definitions that prioritize neuromuscular function as the primary diagnostic domain of sarcopenia [2,11].

Despite the apparent objectivity, reproducibility, and scalability of CT-derived muscle metrics, it is crucial to distinguish these pragmatic advantages from biological or clinical validity. The widespread adoption of imaging-based surrogates has therefore been driven largely by feasibility rather than by concordance with consensus recommendations that prioritize neuromuscular function over morphologic muscle quantity [2,11]. Importantly, the ease with which radiologic skeletal muscle loss can be measured does not mitigate the risk of misclassifying sarcopenia, nor does it justify deviating from function-centered diagnostic frameworks when interpreting clinical relevance or prognostic significance [2,11]. Because these pragmatic drivers operate uniformly across tumor types, they have entrenched a field-wide operational paradigm that prioritizes convenience over conceptual fidelity.

However, contemporary consensus frameworks define sarcopenia as a multidimensional neuromuscular syndrome in which muscle strength and physical performance represent the primary diagnostic domains, with muscle quantity serving as a confirmatory or contextual component rather than the diagnostic entry criterion [2,11,20,22,23,24,25,26,27,28,29,30]. Accordingly, imaging-derived skeletal muscle depletion identified on CT should not be interpreted as a standalone diagnostic criterion for sarcopenia. When reduced skeletal muscle area is used in isolation, it may more accurately represent myopenia—a descriptive term for morphologic muscle depletion rather than a new diagnostic construct—rather than the full clinical syndrome of sarcopenia.

### 4.3. What Is Being “Validated” in Oncology Studies—And What Is Not

CT-derived skeletal muscle indices are well validated for quantifying muscle mass and have demonstrated reproducible associations with survival, postoperative complications, and treatment-related toxicity in selected oncologic contexts [4,5,6,7,8,14,15,16]. However, validation of a surrogate metric should not be conflated with validation of sarcopenia as a clinical syndrome [38,39,40,41].

Radiologic muscle mass does not reliably capture neuromuscular dysfunction, impairments in physical performance, or deterioration in muscle quality, domains that are central to functional decline and treatment vulnerability and that are explicitly prioritized in contemporary consensus definitions [11,17,20]. Importantly, preservation, or even apparent gain, of skeletal muscle area on imaging does not preclude the presence of clinically meaningful sarcopenia, as deficits in strength, power, and muscle quality may emerge independently of, or precede, detectable morphologic change [11,19,20]. Accordingly, what is most often “validated” in oncology is skeletal muscle depletion as a morphologic phenotype, rather than sarcopenia as a multidimensional syndrome.

### 4.4. Consequences of Reductionist Operationalization in Oncology

The persistent reliance on a morphologic definition of sarcopenia carries important scientific and clinical consequences for oncologic research and practice. While CT-derived skeletal muscle indices are valid for quantifying myopenia, the routine elevation of muscle mass loss to a diagnosis of sarcopenia represents a conceptual misalignment, as contemporary consensus definitions explicitly prioritize neuromuscular function over muscle quantity [2,11]. Methodological convenience and widespread availability of imaging, although practical, do not justify deviation from function-centered diagnostic frameworks or serve as a substitute for biological precision.

Across tumor types, exclusive dependence on imaging-derived muscle mass metrics has contributed to marked heterogeneity in reported sarcopenia prevalence and variability in associations with survival, treatment tolerance, and toxicity [18,21]. Even within similar cohorts, differing morphologic thresholds produce widely divergent prevalence estimates and hazard ratios, indicating that operational definition selection substantially shapes reported outcomes.

Evidence of such definition-dependent discordance is illustrated outside oncology as well. Johnston and colleagues directly compared CT-defined sarcopenia with EWGSOP1 and EWGSOP2 definitions in adults evaluated for liver transplantation, demonstrating that CT-defined prevalence (52%) exceeded EWGSOP1-defined (22%) and EWGSOP2-defined (11%) prevalence two- to four-fold [42]. Although conducted in a non-cancer population, this study highlights how substituting morphologic criteria for consensus-based frameworks can substantially alter diagnostic classification.

Comparable variability has been documented in oncology-specific analyses. In a meta-analysis of 52 studies including 16,468 patients with gastrointestinal malignancies, Sun and colleagues reported a pooled sarcopenia prevalence of 33% (95% CI: 28–38%), with estimates ranging from 8% to 77% depending on the criteria applied [43]. Such variability underscores the dominant influence of operational definitions on reported prevalence, often exceeding the influence of tumor biology itself.

More importantly, conflating myopenia with sarcopenia introduces biological imprecision. When muscle strength, physical performance, nutritional status, and inflammatory context are not assessed, sarcopenia may be inadvertently conflated with pathophysiologically distinct entities such as cancer cachexia and malnutrition [22,25,28]. Labeling these conditions as “sarcopenia” based solely on muscle quantity obscures mechanistic differences, complicates prognostic interpretation, and may misdirect therapeutic strategies. Precision in terminology, therefore, serves not merely semantic clarity but clinical and translational rigor.

### 4.5. Representative Oncology Contexts Illustrating the Mismatch

Although radiology-driven operationalization of sarcopenia is widely used in oncology, its limitations are particularly apparent in specific clinical contexts in which neuromuscular dysfunction and functional decline frequently precede or occur independently of measurable muscle mass loss.

In head and neck cancers, treatment-related neuromuscular impairment often emerges early in the disease course. Radiotherapy, concurrent systemic therapy, cranial neuropathy, trismus, dysphagia, and pain can profoundly compromise muscle strength, coordination, and physical performance even when the whole-body skeletal muscle area appears relatively preserved on imaging [33]. Reliance on morphologic criteria alone may therefore underestimate clinically meaningful sarcopenia while conflating treatment-related malnutrition or weight loss with true neuromuscular dysfunction.

In gastrointestinal malignancies—particularly pancreatic, gastric, and colorectal cancers—systemic inflammation, anorexia, and metabolic dysregulation dominate early disease trajectories. These patients frequently develop cancer cachexia, characterized by inflammation-driven muscle wasting that is mechanistically distinct from sarcopenia and poorly responsive to nutritional supplementation or exercise alone [22,23,24,29]. Designating these patients as sarcopenic solely due to diminished muscle mass may obscure cachexia as the principal pathological process and cultivate unrealistic expectations concerning reversibility and the effectiveness of interventions.

In thoracic malignancies, including lung and esophageal cancers, chronic inflammation, hypoxemia, smoking-related comorbidities, and treatment-induced deconditioning contribute to early declines in muscle strength, endurance, and physical performance that may occur in the absence of overt radiologic muscle loss [7,28]. During chemoradiotherapy or immunotherapy, functional deterioration may therefore precede detectable changes in skeletal muscle area, further highlighting the limitations of mass-based definitions.

Importantly, comparable discordances between morphologic muscle depletion and functional impairment have been reported across breast, genitourinary, gynecologic, and hematologic malignancies, indicating that this mismatch reflects a field-wide operational paradigm rather than cancer type-specific biology [18,21,28].

### 4.6. Imaging as a Necessary but Insufficient Component

It is important to emphasize that the preceding critique neither advocates abandoning radiologic assessment of skeletal muscle in oncology nor questions its established clinical relevance. On the contrary, imaging remains indispensable for quantifying skeletal muscle mass, characterizing radiologic markers of muscle quality—including myosteatosis—and monitoring longitudinal changes in body composition throughout cancer therapy and survivorship. CT- and MRI-derived measures provide objective, reproducible, and widely accessible data that have demonstrated consistent associations with survival, treatment-related toxicity, postoperative complications, and functional decline across multiple malignancies. Within oncology workflows, imaging therefore constitutes a powerful platform for structural biomarkers.

CT-derived skeletal muscle indices quantify muscle quantity and provide a reproducible structural metric, most commonly at the L3 vertebral level. However, muscle quantity represents only one domain of the sarcopenia construct. Contemporary consensus frameworks define sarcopenia primarily by impaired muscle strength, with physical performance and muscle quality serving complementary roles. Structural muscle depletion cannot be assumed to reflect neuromuscular integrity or functional capacity, as strength and performance are influenced by neural activation, inflammatory burden, metabolic status, and intramuscular composition. Accordingly, radiologic measures of muscle mass should be interpreted as quantitative descriptors of morphology rather than definitive indicators of the multidimensional syndrome of sarcopenia.

The limitation, therefore, arises only when radiologic muscle metrics are elevated from informative structural descriptors to definitive diagnostic surrogates for sarcopenia as a clinical syndrome. While imaging captures quantitative and compositional features of muscle tissue, it cannot, in isolation, assess neuromuscular strength, contractile function, endurance capacity, or task-specific performance—domains that contemporary consensus frameworks designate as central to the diagnosis of sarcopenia. Exclusive reliance on CT-derived muscle area thus risks conflating myopenia with sarcopenia and obscuring functionally relevant vulnerability, particularly in patients with preserved muscle mass but impaired strength or performance.

**Figure 3:**
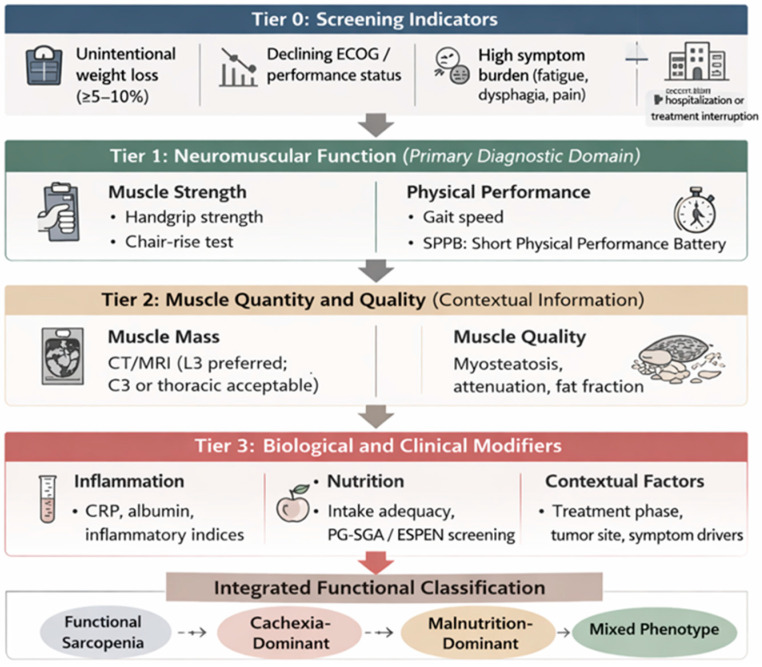
A conceptual multidimensional framework for evaluating sarcopenia and cancer-related muscle dysfunction in oncology. The schematic illustrates a hierarchical approach beginning with clinical risk triggers (Tier 0), followed by neuromuscular function as the primary diagnostic domain (Tier 1). Muscle quantity and quality assessed by imaging are incorporated as contextual information (Tier 2), while biological and clinical modifiers refine phenotypic characterization (Tier 3). The framework culminates in an integrated functional classification reflecting distinct clinical phenotypes.

Radiologic assessment should therefore be regarded as a necessary but insufficient component of sarcopenia evaluation in oncology—embedded within a multidimensional framework that integrates muscle strength, physical performance, muscle quality, inflammatory context, and nutritional status (Figure 3). Such integration preserves the strengths of imaging while restoring conceptual fidelity, improving biological precision, and enhancing translational relevance in cancer care.

### 4.7. Transition to Prognostic and Therapeutic Implications

Clarifying how sarcopenia has been operationalized in oncology is not merely a semantic exercise; it is fundamental to the valid interpretation of prognostic associations, the comparability of interventional trials, and the rational design of supportive care strategies. When imaging-defined muscle depletion (myopenia) is operationally substituted for multidimensional sarcopenia, reported relationships with treatment toxicity, survival, and functional outcomes may primarily reflect the consequences of reduced muscle quantity rather than the broader neuromuscular vulnerability that consensus definitions seek to capture. The following section, therefore, evaluates how differing operational definitions of sarcopenia influence observed prognostic effects and therapeutic inferences across cancer populations, with particular attention to their implications for clinical decision-making and translational relevance.

## 5. Implications of Radiology-Based Single-Metric Definitions of Sarcopenia in Oncology

Despite the availability of multiple international consensus definitions that characterize sarcopenia as a multidimensional neuromuscular disorder—anchored in multidimensional functional assessment—oncology research and clinical practice continue to rely predominantly on radiology-derived measures of skeletal muscle mass as the sole defining criterion [1,2,3,6,8,13,14,15,16]. This persistence is primarily motivated by pragmatic factors, such as the ready availability of CT imaging and the feasibility of retrospective morphometric analysis, rather than by conformity to modern, evidence-based diagnostic standards. The consequence is not merely definitional discordance: reducing sarcopenia to a myopenia-based construct fundamentally shapes prognostic inference, trial interpretation, and supportive care decision-making in oncologic populations. Accordingly, this section examines how radiology-based single-metric definitions influence reported associations with toxicity, survival, functional outcomes, and therapeutic responsiveness across cancer settings.

### 5.1. Artificial Inflation of Sarcopenia Prevalence

Radiology-based single-metric definitions substantially influence reported sarcopenia prevalence in oncology. Meta-analyses and large observational studies consistently demonstrate wide variability—often ranging from below 20% to over 70%—depending on the anatomical landmark selected, cut-off values applied, and characteristics of the study population [4,5,6,8]. This variability reflects not only technical differences but also a fundamental misalignment between morphologic muscle depletion and the multidimensional construct of sarcopenia as defined by contemporary consensus frameworks [2,3,11,12].

In cancer populations, reduced skeletal muscle mass frequently accompanies cachexia, malnutrition, or advanced disease-related weight loss, independent of primary neuromuscular dysfunction. Because reductions in muscle mass do not consistently parallel impairments in strength or performance, reliance on CT-defined thresholds alone captures a biologically heterogeneous group of patients with distinct drivers of vulnerability. The resulting inflation and variability in prevalence complicate inter-study comparisons and obscure the true burden of function-centered sarcopenia across tumor types [5,6,15].

### 5.2. Undervaluation of Reductions in Muscle Strength and Function

Single-metric, mass-based definitions also systematically underrepresent reductions in muscle strength and physical performance—domains consistently shown to predict disability, treatment intolerance, and mortality more robustly than muscle mass alone [10,19,31,35]. Contemporary consensus frameworks explicitly position muscle strength as the primary diagnostic criterion [2,11], yet most oncologic studies continue to operationalize sarcopenia using morphologic surrogates.

In clinical oncology, functional decline may precede measurable muscle atrophy. Treatment-related neurotoxicity, systemic inflammation, hypoxia, and physical inactivity can impair neuromuscular performance even when skeletal muscle mass appears preserved on imaging [6,7,33]. Consequently, exclusive reliance on morphologic criteria may fail to identify early functional vulnerability, particularly in phenotypes such as sarcopenic obesity or pronounced myosteatosis, where strength impairment and muscle quality abnormalities coexist with apparently adequate muscle quantity [20,21,30,34].

### 5.3. Impaired Comparability Across Oncologic Studies

The dominance of CT-based skeletal muscle indices as a surrogate for sarcopenia has also introduced substantial methodological heterogeneity across oncologic research. Differences in vertebral level selection, segmentation techniques, normalization strategies, and threshold derivation limit cross-study comparability and complicate pooled analyses [13,14,15,16,21]. More importantly, studies that equate myopenia with sarcopenia often extrapolate findings to broader concepts of functional reserve or treatment vulnerability without direct assessment of strength or performance.

As a result, meta-analytic estimates of the prognostic impact of “sarcopenia” in oncology frequently reflect definitional inconsistency rather than true biological variability [4,5]. Harmonizing terminology and aligning operational definitions with consensus frameworks would therefore improve interpretability, enhance reproducibility, and reduce artificial heterogeneity across tumor types and treatment settings.

These patterns share a common conceptual origin: the substitution of a structural descriptor for a multidimensional clinical syndrome. When sarcopenia is defined solely through imaging-derived muscle mass, variability extends beyond statistics to influence diagnostic labeling and therapeutic reasoning. The implications of such conflation become particularly evident when sarcopenia overlaps with related entities such as malnutrition and cancer cachexia.

### 5.4. Diagnostic Confusion with Malnutrition and Cancer Cachexia

Radiology-based single-metric definitions of sarcopenia substantially exacerbate diagnostic confusion among sarcopenia, malnutrition, and cancer cachexia. Although all three conditions may present with reduced skeletal muscle mass on imaging, their underlying pathophysiology, reversibility, and clinical implications differ fundamentally [22,23,24,25,26,27,28].

The diagnostic implications of definitional substitution become particularly evident in the context of the international Delphi consensus definition of cancer cachexia proposed by Fearon and colleagues [23]. In this framework, cachexia may be diagnosed by any of three routes: (i) >5% weight loss over the preceding 6 months; (ii) >2% weight loss in individuals with low BMI (<20 kg/m^2^); or (iii) >2% weight loss in the presence of sarcopenia [23]. When “sarcopenia” is operationalized solely as CT-derived low muscle mass, route (iii) becomes vulnerable to inflation because morphologic myopenia can satisfy the sarcopenia criterion without confirmation of neuromuscular dysfunction. This can artificially expand cachexia labeling, inflate prevalence estimates, and propagate downstream distortion in prognostic modeling and interventional trial interpretation.

Cancer cachexia is driven by systemic inflammation and tumor-mediated catabolism, is frequently resistant to nutritional support, and often reflects advanced disease with severely limited physiologic reserve [23,24,28,29]. Malnutrition, in contrast, is primarily substrate-driven and may be partially or fully reversible with timely and targeted nutritional intervention [25,26,27]. Sarcopenia, as defined by contemporary consensus frameworks, represents a distinct neuromuscular disorder characterized by impaired muscle strength and function and may coexist with either cachexia or malnutrition without being reducible to muscle mass loss alone [1,2,3,22].

When patients with cachexia or severe malnutrition are labeled as “sarcopenic” solely on the basis of CT-derived muscle depletion, intervention selection may become misaligned with underlying biology. Exercise-based or rehabilitation-focused approaches may be ineffective or poorly tolerated in the presence of uncontrolled inflammation or metabolic instability, whereas failure to recognize functional sarcopenia in patients with preserved muscle mass may delay appropriate rehabilitation, supportive care optimization, and risk mitigation [6,20,25,30].

### 5.5. Misinterpretation of Therapeutic Efficacy and Toxicity

Perhaps the most consequential implication of myopenia-based sarcopenia definitions lies in the interpretation of oncologic treatment outcomes. Reduced skeletal muscle mass has been consistently associated with increased chemotherapy toxicity, dose reductions, treatment interruptions, and inferior survival across multiple malignancies [6,7,8,14,30]. However, when muscle mass loss alone is used as the defining criterion, these associations become difficult to interpret, as the observed effects may reflect advanced disease burden, systemic inflammation, and nutritional compromise rather than neuromuscular dysfunction in isolation.

This confusion risks misattributing treatment intolerance to sarcopenia itself, rather than to cachexia, malnutrition, or overall disease severity, and may foster unrealistic expectations regarding the efficacy of targeted interventions. Interventional trials that aim to modify “sarcopenia” without stratification by functional status, inflammatory burden, or nutritional adequacy are therefore prone to equivocal or misleading results. From a clinical perspective, such misclassification may expose patients to ineffective or burdensome interventions, contribute to avoidable toxicity, and impose unnecessary financial and resource-related strain without delivering meaningful benefit [6,7,25,30].

### 5.6. Consequences for Clinical Decision-Making and Trial Design in Oncology

Beyond the epidemiologic, diagnostic, and prognostic consequences outlined above, reliance on radiology-based single-metric definitions of sarcopenia has direct, clinically meaningful implications for treatment decision-making and research design in oncology. Increasingly, sarcopenia status is used to guide chemotherapy dosing, treatment intensity, perioperative risk stratification, and eligibility for supportive or prehabilitative interventions. When sarcopenia is operationalized solely as low skeletal muscle mass, downstream decisions risk being grounded in an imprecise, biologically heterogeneous construct.

In routine clinical practice, patients labeled as sarcopenic based on imaging alone may undergo unwarranted treatment modification, including dose reductions, regimen alterations, or premature treatment discontinuation. Systemic inflammation, malnutrition, or active cachexia are often the leading causes of treatment intolerance, not just neuromuscular dysfunction. Conflating these entities under a single morphologic label risks prematurely attributing toxicity to an inherently non-modifiable condition, thereby obscuring potentially reversible contributors such as inadequate nutritional intake or uncontrolled inflammatory burden [23,24,25,26,30].

From a research perspective, myopenia-based sarcopenia definitions introduce substantial heterogeneity into interventional trials. Exercise, nutritional, or multimodal interventions targeting “sarcopenia” frequently enroll patients with divergent biological phenotypes, including functional sarcopenia, cachexia, and advanced malnutrition. This phenotypic mixing systematically dilutes treatment effects, increases variance, and contributes to neutral or inconclusive trial outcomes despite sound mechanistic rationale. Consequently, potentially effective interventions may be dismissed as ineffective due to inappropriate patient selection rather than true lack of efficacy [6,25,27].

Moreover, single-metric definitions impede the development of precision supportive oncology. Patients with preserved muscle mass but profound reductions in muscle strength or marked myosteatosis—such as those with sarcopenic obesity—may be excluded from targeted interventions. In contrast, patients with advanced cachexia may be inappropriately assigned to exercise-based strategies that are poorly tolerated or biologically implausible. Such mismatches undermine both patient outcomes and efficient resource allocation.

Collectively, these issues underscore that definitional choices are not merely semantic or methodological but carry tangible consequences for patient care, trial validity, and translational progress. Adoption of multidimensional assessment strategies that integrate muscle strength, muscle quality, inflammatory status, and nutritional context is therefore essential to support rational clinical decision-making and to enable biologically coherent intervention trials in oncology.

## 6. Toward a Multidimensional, Oncology-Adapted Approach to Sarcopenia

The preceding sections demonstrate that many of the apparent inconsistencies, contradictions, and controversies surrounding sarcopenia in oncology stem not from conceptual ambiguity but from incomplete and reductionist operationalization of well-established consensus principles in cancer-specific contexts. Contemporary frameworks consistently define sarcopenia as a multidimensional neuromuscular disorder, prioritizing muscle strength, physical performance, and muscle quality over muscle mass alone [2,11,12]. Nonetheless, oncological research and clinical practice continue to depend excessively on radiology-derived indicators of muscle mass as sole diagnostic proxies. It is unnecessary to redefine sarcopenia to resolve this disconnect; instead, existing consensus principles should be adapted for oncology, integrating functional, biological, and clinical dimensions while remaining aligned with the practicalities of cancer care.

### 6.1. Principles for Oncology-Appropriate Sarcopenia Assessment

Several core principles should guide an oncology-appropriate approach to assessing sarcopenia. First, sarcopenia is fundamentally a state of neuromuscular vulnerability, not a deficit of muscle quantity. Accordingly, impairments in muscle strength and physical performance—rather than reductions in muscle mass alone—represent the most clinically meaningful abnormalities and the strongest determinants of adverse outcomes [2,11,19,31]. While muscle mass remains an informative descriptor of body composition, it neither defines sarcopenia nor reliably identifies patients at greatest functional or treatment-related risk when interpreted in isolation.

Second, sarcopenia assessment in oncology must be explicitly context-sensitive. Systemic inflammation, nutritional compromise, tumor burden, and treatment-related toxicities frequently coexist and dynamically interact, modulating both neuromuscular function and responsiveness to intervention [6,23,24,25,26]. Failure to account for this biological context risks misclassification and misdirected therapeutic prioritization.

Third, feasibility must be treated as an essential design constraint rather than a secondary consideration. Sarcopenia assessment strategies should balance biological rigor with pragmatic realities, recognizing that time, resources, and patient tolerance vary substantially across disease sites, treatment phases, and care settings. Accordingly, oncology-adapted sarcopenia assessment requires scalable, flexible approaches rather than rigid or idealized protocols.

Taken together, these principles are closely aligned with contemporary international consensus frameworks, such as the EWGSOP2, SDOC, and the GLIS. These frameworks emphasize strength-centered diagnosis, contextual interpretation, and adaptable implementation, rather than single-metric definitions [2,11,12].

### 6.2. A Tiered and Feasible Assessment Strategy in Oncology Practice

Rather than relying on a singular, inflexible diagnostic algorithm, an oncology-adapted approach to sarcopenia assessment should adopt a pragmatic, tiered workflow that accommodates the inherent heterogeneity of clinical settings, disease trajectories, retrospective datasets, and resource availability [11,20,27,28] (Table 3). In contemporary oncology practice, assessment may appropriately begin with CT-derived morphologic evaluation, leveraging the routine availability of cross-sectional imaging throughout the cancer care continuum. Within this stepwise workflow, individuals demonstrating radiologically defined muscle depletion may subsequently undergo functional screening—such as handgrip dynamometry, chair-rise testing, or gait speed assessment—when feasible, thereby facilitating distinction between multidimensional sarcopenia and isolated morphologic muscle loss [10,19,31].

These functional assessments are inexpensive, reproducible, and consistently associated with adverse outcomes—including treatment intolerance, functional decline, and mortality—across diverse oncologic populations [4,5,6,7,8]. CT-derived skeletal muscle metrics remain clinically informative structural biomarkers but should not be interpreted as definitive indicators of sarcopenia. Sarcopenia remains a multidimensional neuromuscular syndrome that requires integration of functional assessment for accurate phenotypic characterization.

Subsequent contextual interpretation incorporating muscle quality, nutritional status, inflammatory burden, longitudinal weight trajectory, and cachexia-related features may further refine phenotypic classification and clinical interpretation. At an intermediate tier, functional evaluation may therefore be complemented by imaging-based assessment of muscle quantity and quality using routinely acquired CT or MRI examinations. Particular attention should be directed toward muscle quality indices such as myosteatosis, which have demonstrated prognostic relevance in oncology independent of muscle cross-sectional area alone [20,21,30]. Concurrent nutritional screening and longitudinal assessment of body weight may further assist in distinguishing sarcopenia from malnutrition or evolving cachexia [25,26,27].

In research-intensive environments, or among patients at elevated risk of treatment-related toxicity, more comprehensive multidimensional assessment strategies may be appropriate. Such approaches may integrate muscle strength, physical performance, quantitative and qualitative muscle imaging, inflammatory biomarkers, nutritional status, and broader indicators of clinical vulnerability. This layered stratification may improve phenotypic precision, strengthen the interpretability of interventional research, and support biologically coherent supportive oncology strategies while preserving feasibility across diverse clinical environments. Importantly, such an approach remains scalable across retrospective datasets, routine oncology workflows, and resource-variable practice settings.

### 6.3. Implications for Clinical Trials and Translational Research

The adoption of multidimensional sarcopenia assessment strategies has substantial implications for clinical trials and translational research in oncology. Interventional studies targeting sarcopenia have frequently yielded neutral or heterogeneous results, in part because study populations are often defined using muscle mass-based criteria that aggregate biologically distinct phenotypes, including functional sarcopenia, cancer cachexia, and severe malnutrition [6,25,27]. This phenotypic mixing dilutes treatment effects, increases variance, and obscures the detection of mechanistic signals.

In addition to definitional aggregation, methodological practices may further contribute to instability. Skeletal muscle mass, muscle strength, and physical performance are continuous and interrelated biological variables. Dichotomizing CT-derived muscle indices at arbitrary thresholds can obscure clinically meaningful risk gradients and amplify instability near cut-off points, particularly when threshold selection varies across cohorts. Modeling muscle measures as continuous variables, incorporating non-linear relationships where appropriate, and integrating complementary constructs such as mass and strength may preserve prognostic information while reducing artificial heterogeneity. Within such an analytic framework, imaging-based myopenia retains clinical and translational relevance when interpreted as a structural biomarker rather than as a standalone diagnostic surrogate.

Improved stratification—specifically distinguishing neuromuscular dysfunction–dominant sarcopenia from inflammation- or substrate-driven muscle loss—has the potential to enhance signal detection, reduce heterogeneity, and improve the interpretability and reproducibility of trial outcomes. Beyond intervention trials, reframing sarcopenia assessment also refines the interpretation of treatment-related toxicity and therapeutic efficacy. By disentangling neuromuscular vulnerability from inflammation-driven catabolism or nutritional deficiency, clinicians and researchers can more accurately identify which patients are most likely to benefit from exercise-based rehabilitation, nutritional optimization, anti-inflammatory strategies, or multimodal supportive care approaches [23,24,25,26,30]. Such alignment between biological phenotypes and intervention targets is central to advancing precision supportive oncology and translating mechanistic insight into clinically meaningful benefit.

Beyond biological precision, phenotypic clarity may also have implications for resource allocation. Structured exercise programs, nutritional supplementation, pharmacologic agents, and multimodal supportive interventions require dedicated personnel, infrastructure, and financial investment. When biologically heterogeneous phenotypes are grouped under a single sarcopenia label, preventive or therapeutic maneuvers may be deployed without clear mechanistic alignment, reducing efficiency and potentially contributing to avoidable healthcare expenditure. More accurate stratification may therefore enhance not only signal detection and clinical targeting but also the responsible deployment of supportive oncology resources.

### 6.4. Sarcopenia Within the Spectrum of Cancer-Related Muscle Failure

In the oncologic context, sarcopenia rarely exists in isolation. Rather, it represents one component within a broader spectrum of cancer-related muscle failure, encompassing cachexia and malnutrition as overlapping yet biologically distinct entities [22,23,24,25,26,27,36]. Although these conditions may converge phenotypically as reduced muscle mass, they differ fundamentally in their dominant pathogenic drivers, temporal evolution, reversibility, and responsiveness to intervention. Accordingly, the clinically relevant question is not whether a patient satisfies a singular diagnostic label, but rather which pathological processes predominate at a given time and how they interact across the cancer trajectory.

Conceptualizing sarcopenia as a state of neuromuscular vulnerability that may coexist with inflammation-driven catabolism or substrate deficiency provides a more clinically actionable framework. This perspective preserves the conceptual coherence of sarcopenia as a functionally defined syndrome while simultaneously recognizing the biological complexity of cancer-associated muscle dysfunction. Importantly, it enables clinicians and researchers to distinguish patients in whom functional impairment may be amenable to rehabilitation or neuromuscular interventions from those in whom systemic inflammation, metabolic instability, or nutritional compromise constitute the dominant pathological constraint.

The widespread adoption of CT-derived skeletal muscle assessment in oncology has largely reflected pragmatic clinical and methodological considerations rather than intentional deviation from contemporary sarcopenia frameworks. Cross-sectional imaging is routinely obtained throughout the oncologic continuum for staging, radiotherapy planning, treatment response assessment, and surveillance, rendering skeletal muscle quantification readily accessible in both retrospective datasets and routine clinical practice without additional patient burden. By contrast, standardized assessments of muscle strength, physical performance, muscle quality, nutritional status, and inflammatory context are infrequently incorporated into routine oncology workflows, particularly in retrospective cohorts. Consequently, CT-derived skeletal muscle metrics became operationally attractive because they are reproducible, scalable, anatomically validated, and consistently associated with oncologic risk stratification, including treatment-related toxicity, postoperative complications, and survival [4,5,6,7,8,13,14,15,16,18,21].

Importantly, acknowledging these practical realities does not diminish the conceptual limitations of defining sarcopenia solely through muscle mass. Rather, it underscores why radiologic muscle assessment should be interpreted as a clinically valuable structural biomarker—more precisely reflecting myopenia—particularly in settings where comprehensive functional assessment is not feasible. Within this framework, greater terminological precision and multidimensional interpretation may strengthen, rather than invalidate, the substantial body of prognostic oncology literature derived from CT-based muscle assessment [4,5,6,7,8,13,14,15,16,18,21].

Adopting a spectrum-based framework, therefore, enables more individualized assessment and intervention, supports biologically coherent trial design, and aligns sarcopenia evaluation with precision supportive oncology. In contrast to reductionist single-metric classifications that artificially fragment patients into mutually exclusive categories, this model explicitly recognizes coexistence, transition, and overlap as defining features of cancer-related muscle failure—features that may otherwise remain systematically underrecognized or obscured.

### 6.5. Future Directions and Research Priorities

Future oncology research must advance decisively toward prospective, multidimensional study designs that integrate functional, morphologic, and metabolic domains, rather than continuing to rely on isolated surrogates of muscle mass. Core priorities include the prospective validation of pragmatic, scalable functional assessment tools across diverse cancer populations; systematic incorporation of muscle quality metrics, such as myosteatosis, into standardized imaging workflows; and phenotype-driven interventional trials that more precisely align therapeutic strategies with underlying biological vulnerability. Equally important is moving beyond the use of radiologic muscle metrics as a principal endpoint in sarcopenia-focused oncology research, as isolated morphologic assessment incompletely captures neuromuscular dysfunction and may inadequately reflect clinically meaningful physiologic vulnerability.

Future efforts should also emphasize greater terminologic precision and more refined clinical phenotyping in oncology body-composition research. Distinguishing CT-defined muscle depletion/myopenia from multidimensional sarcopenia may improve diagnostic clarity, reduce biological misclassification, and enhance interpretability and comparability across studies. The development of more integrated phenotyping frameworks capable of differentiating sarcopenia, cachexia, malnutrition, sarcopenic obesity, and overlapping cancer-related muscle-failure states may improve prognostic modeling, facilitate mechanism-aligned intervention selection, and strengthen translational interpretation in supportive oncology research.

Importantly, such approaches should remain scalable and adaptable across retrospective datasets, prospective clinical trials, and resource-variable oncology practice environments. Because comprehensive neuromuscular assessment may not always be feasible in real-world clinical settings, future frameworks should prioritize pragmatic integration rather than idealized diagnostic completeness. Within this context, CT-derived muscle metrics should continue to be recognized as clinically meaningful structural biomarkers while being interpreted within broader multidimensional and context-sensitive assessment models.

Critically, meaningful progress in this field depends less on generating additional competing definitions than on the rigorous, context-sensitive implementation of existing consensus frameworks within oncology-specific settings. By systematically integrating functional assessment, imaging-derived muscle characteristics, and nutritional and inflammatory context, clinicians and investigators may more accurately characterize physiologic reserve, improve diagnostic precision, minimize biological ambiguity, and support more rational, mechanism-aligned, and genuinely patient-centered supportive oncology strategies [2,11,12].

## 7. Limitations

Several limitations inherent to the present review warrant acknowledgment. Foremost, this work was conceived as a narrative and conceptual synthesis rather than a systematic evidence synthesis; accordingly, both literature selection and interpretive approach were deliberately illustrative and hypothesis-driven rather than exhaustive or quantitatively aggregated. Second, the extant oncology literature is characterized by substantial heterogeneity across tumor types, treatment settings, imaging methodologies, diagnostic thresholds, and outcome definitions, which may constrain direct cross-study comparability and contribute to variability in reported sarcopenia prevalence and prognostic associations.

Furthermore, many retrospective oncology datasets are limited by the absence of standardized assessments of muscle strength, physical performance, muscle quality, nutritional status, and inflammatory milieu. This methodological constraint has historically fostered reliance on CT-derived morphologic muscle metrics within oncology research and has impeded retrospective application of contemporary multidimensional consensus frameworks. Lastly, the ongoing evolution of international sarcopenia definitions and the continued development of regionally adapted diagnostic criteria may continue to influence interpretation and harmonization of future oncology body-composition investigations.

## 8. Conclusions

Sarcopenia has become recognized as a clinically important and prognostically relevant construct in oncology; however, its operationalization in cancer research has frequently diverged from contemporary consensus definitions. Although international frameworks define sarcopenia as a multidimensional neuromuscular syndrome characterized by deficits in functional and qualitative domains, oncologic investigations have frequently used CT-derived skeletal muscle mass as a single diagnostic proxy. This methodological divergence has contributed to definitional ambiguity, heterogeneity in prevalence estimates, and inconsistencies in the interpretation of treatment-related outcomes across diverse cancer populations.

Importantly, this analysis does not seek to diminish the established clinical utility of CT-derived skeletal muscle assessment. Radiologically defined muscle depletion—more precisely termed myopenia—remains a reproducible, scalable, and clinically informative structural biomarker, with consistent prognostic relevance documented across malignancies. Nevertheless, myopenia constitutes a morphologic phenotype, whereas sarcopenia represents a higher-order neuromuscular syndrome characterized by functional impairment and, within contemporary frameworks, graded severity. Conflation of these constructs risks biological misclassification and may obscure clinically meaningful distinctions among sarcopenia, cachexia, malnutrition, and related cancer-associated muscle failure states.

Accordingly, future oncology research and clinical practice should prioritize terminological precision, refined clinical phenotyping, and scalable context-sensitive evaluation strategies that integrate functional evaluation, imaging-derived muscle characteristics, and relevant nutritional and inflammatory parameters whenever feasible. Progress in the field depends less on proliferating novel or competing definitions than on the rigorous and context-sensitive implementation of existing consensus frameworks within oncology-specific contexts.

By preserving the clinical value of radiologic muscle assessment while restoring conceptual fidelity to contemporary sarcopenia frameworks, oncology may achieve more coherent risk stratification, reduce biological misclassification, enhance translational interpretability, and support more rational, mechanism-aligned, and patient-centered supportive care strategies.

## Figures and Tables

**Table 1:** Evolution of international consensus definitions of sarcopenia.

Consensus Group (Year)	Primary Diagnostic Focus	Core Diagnostic Components	Key Diagnostic Thresholds/Concepts	Major Conceptual Contribution	Key Limitations/Considerations
EWGSOP1 (2010)	Combined structure and function	Muscle mass plus muscle strength or physical performance	Low appendicular lean mass (DXA); gait speed <0.8 m/s	Introduced the structural–functional model; first widely adopted clinical definition	Emphasis on muscle mass; potential misclassification in small-bodied, obese, or cachectic populations; limited oncology-specific validation
IWGS (2011)	Mobility-centered	Gait speed plus appendicular lean mass	Gait speed <1.0 m/s; low ALM/height^2^	Framed sarcopenia as a mobility-related disorder; emphasized early functional decline	May under-detect weakness in individuals with preserved gait speed, including sarcopenic obesity
FNIH (2014)	Empirically derived weakness	Grip strength (primary) plus ALM adjusted for BMI	Grip strength <26 kg (men), <16 kg (women); ALM/BMI <0.789 (men), <0.512 (women)	Established data-driven thresholds; reinforced primacy of strength over mass	Developed in predominantly North American cohorts; debated applicability in oncology and non-Western populations
EWGSOP2 (2019)	Strength-first hierarchical model	Muscle strength → muscle mass → physical performance	Staged diagnosis: probable, confirmed, severe sarcopenia	Formalized strength as entry criterion; introduced diagnostic staging	Continued requirement for mass confirmation; cut-offs require contextual adaptation
AWGS2 (2020)	Strength-first with regional adaptation	Muscle strength, muscle mass, physical performance	Asian-specific cut-offs for grip strength, gait speed, and muscle mass	Addressed ethnic and anthropometric variability; improved regional applicability	Limited validation outside Asian populations; variability in implementation
SDOC (2020)	Strength-centered	Muscle strength alone (primary)	Grip strength as essential diagnostic criterion	Demonstrated prognostic superiority of strength over mass; proposed mass-optional framework	Does not provide comprehensive staging framework
GLIS (2024)	Multidimensional harmonization	Muscle strength, muscle mass, muscle quality	Emphasis on adaptable thresholds rather than fixed universal cut-offs; inclusion of myosteatosis	Distinguished diagnostic from prognostic constructs; integrated muscle quality; promoted global harmonization	Ongoing validation; implementation complexity in resource-constrained settings

**Abbreviations:** EWGSOP1, European Working Group on Sarcopenia in Older People (2010); IWGS, International Working Group on Sarcopenia; FNIH, Foundation for the National Institutes of Health Sarcopenia Project; EWGSOP2, European Working Group on Sarcopenia in Older People (2019); AWGS2, Asian Working Group for Sarcopenia (2019 update); SDOC, Sarcopenia Definitions and Outcomes Consortium; GLIS, Global Leadership Initiative on Sarcopenia; ALM, appendicular lean mass; BMI, body mass index; DXA, dual-energy X-ray absorptiometry.

**Table 2:** Oncology-specific patterns distinguishing sarcopenia, cancer cachexia, and malnutrition [2,11,20,22,23,24,25,26,27,28,29,30].

Domain	Sarcopenia	Cancer Cachexia	Malnutrition
Primary driver	Neuromuscular dysfunction related to aging, inactivity, or treatment-associated stress	Persistent systemic inflammation and tumor-driven catabolism	Inadequate energy and protein intake
Typical oncologic contexts	Head and neck cancer (post-radiotherapy neuromuscular injury); lung cancer with early functional decline; sarcopenic obesity	Pancreatic, gastric, lung, and advanced gastrointestinal cancers with high inflammatory burden	Esophageal cancer with dysphagia; gastrointestinal obstruction; treatment-related anorexia
Inflammatory state	Absent or low-grade	Prominent and sustained (elevated CRP and pro-inflammatory cytokines)	Usually absent or low-grade
Weight loss	May be absent	Common and progressive	Common; intake-related
Muscle strength	Reduced (primary abnormality)	Reduced (secondary to catabolic drive)	Reduced (secondary to substrate deficiency)
Physical performance	Impaired	Frequently impaired	Variable; may improve with nutritional repletion
Muscle mass (CT/MRI)	Normal or reduced	Reduced	Reduced
Muscle quality (myosteatosis)	Frequently present	Frequently present	Variable
Response to nutrition alone	Partial improvement	Limited unless inflammatory drivers are addressed	Often favorable if implemented early
Response to exercise	Beneficial	Limited unless inflammation is controlled	Beneficial once nutritional status improves
Common misclassification in oncology	Under-recognized when assessment relies on muscle mass alone	Frequently labeled as “sarcopenia”	Frequently labeled as “sarcopenia”
Clinical consequence of misclassification	Unrecognized functional vulnerability and treatment risk	Ineffective exercise-only strategies	Delayed nutritional intervention
Assessment priorities	Muscle strength, physical performance, muscle quality	Inflammatory markers, weight trajectory, muscle strength	Nutritional intake, weight trajectory, muscle strength
Therapeutic emphasis	Exercise-based rehabilitation with nutritional support	Multimodal management (anti-inflammatory, metabolic, nutrition ± exercise)	Nutritional optimization with functional support as needed

**Abbreviations:** CT, computed tomography; MRI, magnetic resonance imaging; CRP, C-reactive protein. **Note:** These conditions may coexist within individual patients. The table summarizes predominant biological and clinical features and is intended to aid conceptual differentiation rather than imply mutually exclusive diagnoses.

**Table 3:** Minimal Core Dataset for Multidimensional Assessment of Sarcopenia in Oncology.

Domain	Assessment Component	Preferred Tools/Metrics	Clinical/Research Rationale	Implementation Level
Muscle Strength	Upper-limb strength	Handgrip strength (dynamometer; best of 2–3 attempts)	Primary diagnostic domain in EWGSOP2, SDOC, and GLIS; consistently associated with disability, treatment toxicity, and mortality	Minimum
	Lower-limb strength	Five-times sit-to-stand (chair-rise) test	Reflects functional power and treatment-related neuromuscular decline; complements grip strength	Recommended
Physical Performance	Mobility	Gait speed (4 m or 6 m walk)	Sensitive to early functional decline; prognostic across multiple cancer types	Minimum (≥1 performance test)
	Global performance	Short Physical Performance Battery (SPPB)	Enables severity staging and longitudinal monitoring	Recommended
Muscle Quantity (Imaging)	Skeletal muscle mass	CT- or MRI-derived cross-sectional area or index (L3 preferred; C3 or thoracic levels acceptable if abdominal imaging unavailable)	Quantifies myopenia; structural and contextual component rather than standalone diagnostic criterion	Minimum (if imaging available)
Muscle Quality (Imaging)	Myosteatosis/muscle density	CT muscle attenuation (HU) or MRI fat fraction	Independent prognostic marker; refines risk stratification beyond muscle area alone	Recommended
Body-Weight Trajectory	Recent weight change	Percentage weight loss over 3–6 months	Distinguishes sarcopenia from cachexia and malnutrition phenotypes	Minimum
Nutritional Status	Screening	PG-SGA, MUST, or ESPEN-aligned tools	Identifies substrate deficiency and informs nutritional intervention	Minimum
	Intake adequacy	Energy and protein intake estimation	Differentiates malnutrition-dominant from neuromuscular phenotypes	Recommended
Inflammatory/Metabolic Context	Systemic inflammation	CRP ± albumin (or CRP-based indices)	Supports identification of cachexia-dominant phenotypes	Recommended
Clinical Context	Treatment phase	Baseline, on-treatment, post-treatment	Phenotype expression and reversibility vary across the cancer trajectory	Minimum
	Symptom drivers	Dysphagia, trismus, pain, neuropathy, fatigue	Explains discordance between muscle mass and function, particularly in head and neck and thoracic malignancies	Recommended
Reporting Standards	Definition transparency	Explicit citation of diagnostic framework and cut-offs	Reduces heterogeneity and improves cross-study comparability	Minimum
	Missing data handling	Predefined analytic strategy	Mitigates bias in functional and imaging assessments	Recommended

**Abbreviations**: CT, computed tomography; MRI, magnetic resonance imaging; EWGSOP2, European Working Group on Sarcopenia in Older People (2019); SDOC, Sarcopenia Definitions and Outcomes Consortium; GLIS, Global Leadership Initiative on Sarcopenia; HU, Hounsfield units; PG-SGA, Patient-Generated Subjective Global Assessment; MUST, Malnutrition Universal Screening Tool; ESPEN, European Society for Clinical Nutrition and Metabolism; CRP, C-reactive protein. Note: This table proposes a minimal, scalable core dataset rather than a rigid diagnostic algorithm. Components may be implemented in a tiered fashion depending on clinical context, disease site, treatment phase, and resource availability. Muscle mass and imaging-derived metrics are intended to contextualize neuromuscular findings and should not be used as standalone diagnostic proxies for sarcopenia. **Note**: This table proposes a scalable core dataset rather than a prescriptive diagnostic algorithm. Components may be implemented in a tiered manner according to clinical context, disease site, treatment phase, and resource availability. Imaging-derived muscle metrics are intended to contextualize neuromuscular findings and should not be used as standalone diagnostic surrogates for sarcopenia.

## Data Availability

No new data were created or analyzed in this study. Data sharing is not applicable to this article.
